# Health services use by children identified as heterozygous hemoglobinopathy mutation carriers via newborn screening

**DOI:** 10.1186/s12887-021-02751-8

**Published:** 2021-07-01

**Authors:** Sara D. Khangura, Beth K. Potter, Christine Davies, Robin Ducharme, A. Brianne Bota, Steven Hawken, Kumanan Wilson, Maria D. Karaceper, Robert J. Klaassen, Julian Little, Ewurabena Simpson, Pranesh Chakraborty

**Affiliations:** 1 School of Epidemiology and Public Health, University of Ottawa, 415 Smyth Road, Ottawa, Ontario K1H 8M8 Canada; 2 Newborn Screening Ontario, Ottawa, Ontario Canada; 3 ICES, University of Ottawa campus, Ottawa, Ontario Canada; 4 Ottawa Hospital Research Institute, Ottawa, Ontario Canada; 5 Children’s Hospital of Eastern Ontario, Ottawa, Ontario Canada; 6 Department of Medicine, University of Ottawa, Ottawa, Ontario Canada; 7 Bruyère Research Institute, Ottawa, Ontario Canada; 8 Department of Pediatrics, University of Ottawa, Ottawa, Ontario Canada

**Keywords:** Sickle cell disease, Sickle cell trait, Newborn screening, Carrier status, Health services utilization

## Abstract

**Background:**

Newborn screening (NBS) for sickle cell disease incidentally identifies heterozygous carriers of hemoglobinopathy mutations. In Ontario, Canada, these carrier results are not routinely disclosed, presenting an opportunity to investigate the potential health implications of carrier status. We aimed to compare rates of health services use among children identified as carriers of hemoglobinopathy mutations and those who received negative NBS results**.**

**Methods:**

Eligible children underwent NBS in Ontario from October 2006 to March 2010 and were identified as carriers or as screen-negative controls, matched to carriers 5:1 based on neighbourhood and timing of birth. We used health care administrative data to determine frequencies of inpatient hospitalizations, emergency department (ED) visits, and physician encounters through March 2012, using multivariable negative binomial regression to compare rates of service use in the two cohorts. We analyzed data from 4987 carriers and 24,935 controls.

**Results:**

Adjusted incidence rate ratios (95% CI) for service use in carriers versus controls among children < 1 year of age were: 1.11 (1.06–1.17) for ED visits; 0.97 (0.89–1.06) for inpatient hospitalization; and 1.02 (1.00–1.04) for physician encounters. Among children ≥1 year of age, adjusted rate ratios were: 1.03 (0.98–1.07) for ED visits; 1.14 (1.03–1.25) for inpatient hospitalization and 0.92 (0.90–0.94) for physician encounters.

**Conclusions:**

While we identified statistically significant differences in health services use among carriers of hemoglobinopathy mutations relative to controls, effect sizes were small and directions of association inconsistent across age groups and health service types. Our findings are consistent with the assumption that carrier status is likely benign in early childhood.

## Background

Hemoglobinopathies, including sickle cell disease and related disorders, are a group of single gene diseases with an autosomal recessive inheritance pattern. These diseases are characterized by a heterogeneous range of symptoms related to the risk of hemolysis due to mutations that cause malformation or reduced synthesis of hemoglobin or its subunits. Hemoglobinopathies are now the most common life-threatening, monogenic group of disorders worldwide [1, 2]. An estimated 3.0% of people in the Americas are carriers of a hemoglobinopathy variant that can contribute to clinically recognized disease [2].

Population-wide newborn screening (NBS) for sickle cell disease has been implemented in many jurisdictions in recent decades [3, 4]. Pre-symptomatic identification of sickle cell disease by NBS facilitates early initiation of preventive interventions to reduce the occurrence and consequences of acute crises. These interventions include, for example, penicillin prophylaxis, pneumococcal vaccination, and counselling of parents to manage symptoms and monitor for signs of acute crises [5]. In addition to identifying children with sickle cell disease, NBS also incidentally identifies children with other hemoglobinopathies such as alpha- and beta-Thalassemia, as well as a much larger group of children in the screened population who are unaffected carriers of pathogenic hemoglobin mutations [6–9]. In this way, sickle cell disease screening differs from NBS for most other inherited diseases that are screening targets, for which only a fraction of carriers are identified in the screening process.

While generally considered to be clinically benign, sickle cell trait (i.e. carrier status for the HbS variant) has been associated in some studies with a risk of sickling symptoms in particular circumstances, for example during extreme physical exertion and/or increased altitude [10, 11]. While uncertainty remains as to the nature, severity, and extent of the risk associated with carrier status for sickle cell disease [12], particularly for the general population of carriers [13–17], it bears relevance to a broader debate concerning the disclosure to families of carrier results incidentally identified by NBS [18, 19]. Specifically, while most NBS programs have opted for disclosure of all carrier status results to families, some have argued that this may impose undue burden, requiring families to be informed of incidentally generated and clinically ambiguous findings when they may neither need nor prefer to know [20]. Studies of preferences among families regarding the disclosure of carrier status identified by NBS [21, 22], including preferences regarding communication of this information to the children themselves once they have reached adolescence [23], have yielded inconsistent findings [24]. Our research group previously conducted surveys and focus groups among healthcare providers, parents of carrier infants and new parents to evaluate consumer and provider attitudes to inform policies on carrier status disclosure. Responses were mixed with most new parents considering the carrier status to be a benign finding, however uncertainty persisted among some parents and disclosure preferences varied [24]. There is also a lack of evidence regarding the effect of different approaches to managing the disclosure of such carrier results [25].

Ontario became the first jurisdiction in Canada to implement universal NBS for sickle cell disease in 2006. As with most NBS programs across the world, heterozygous carrier status for hemoglobinopathy mutations is not a target of screening in Ontario, but is identified incidentally; approximately 1% of the 140,000 newborns who receive NBS each year in Ontario are identified as carriers of HbS, HbC, Hb D, or Hb E. These carrier results are made available only to those families who formally request this information through their family physician or directly from Newborn Screening Ontario; i.e., the carrier results are not otherwise disclosed proactively. This carrier disclosure approach, which has been called the “positive disclosure model” [26], has been in place in Ontario since 2010 and stands in contrast to the default-disclosure approach taken by many other NBS programs [20, 25]. Information about the ability and process for parents to request carrier results is included in the general information about screening provided to parents at the time of screening, in education provided to birthing centres and midwives, and on the program website. To date, only a small proportion of carriers (e.g., approximately 1% of carriers identified in 2017, personal communication, Newborn Screening Ontario) have requested carrier status information from Newborn Screening Ontario [26]. In light of this low uptake, more targeted communication methods have been contemplated (e.g. communications via community associations, further education for primary care providers, and post-screening direct messages to parents) but not implemented to date. Thus, for the vast majority of children in Ontario who are identified as hemoglobinopathy carriers, this information is not known to their families (aside from those few who request the NBS results or who may have received testing by another means due to family history or affected siblings).

Given Newborn Screening Ontario’s current approach to the disclosure of hemoglobinopathy carrier status and the uncertainty surrounding the clinical significance of carrier status, we sought to use population-based health care administrative data to compare health services use among Ontario children identified by NBS as hemoglobinopathy carriers and those with negative NBS results. Ontario has a large population (over 14 million residents and approximately 140,000 live births/year), in which virtually all individuals are covered by universal health insurance. This allows researchers to link provincial datasets, yielding near-complete population coverage for NBS results, emergency department (ED) visits, hospitalizations and physician encounters. Using these linked datasets, we aimed to compare longitudinal health services use between carriers and matched population controls in a 3.5-year birth cohort.

## Materials and methods

The study protocol was approved by the Ottawa Health Science Network Research Ethics Board (protocol # 2010689-01H).

### Cohort and data assembly

Our retrospective cohort study used routinely-collected health administrative data housed at ICES (formerly known as the Institute for Clinical Evaluative Sciences). ICES is an independent, non-profit research institute funded by an annual grant from the Ontario Ministry of Health and Long-Term Care (MOHLTC). As a prescribed entity under Ontario’s privacy legislation, ICES is authorized to collect and use health care data for the purposes of health system analysis, evaluation and decision support. Secure access to these data is governed by policies and procedures that are approved by the Information and Privacy Commissioner of Ontario.

Eligible children were Ontario residents who underwent NBS between October 2006 and March 2010 (nearly all newborns born in the province during that time period), and were either identified as heterozygous carriers of the HbS (sickle cell trait), HbC, HbD, or HbE variants; or, screened negative for all hemoglobinopathy variants and all conditions on the Ontario NBS panel. Children also had to be eligible for coverage by the Ontario Health Insurance Plan (OHIP) (> 95% of the population).

Confirmed NBS results for each included child were securely linked with demographic and health services use data. The Registered Person’s Database includes vital statistics on OHIP-eligible Ontarians, including dates of birth and death, and residential postal code [27]. Health services outcomes included ED visits, inpatient hospitalizations and physician encounters from birth until March 31, 2012, unless a child’s data were censored earlier due to death or a move away from Ontario. ED visits and inpatient hospitalizations were sourced from the National Ambulatory Care Reporting System (NACRS) and Discharge Abstracts Database (DAD), respectively, both of which are assembled and maintained by the Canadian Institute for Health Information (CIHI) [27]. Physician encounters were identified from OHIP physician billing data, where one encounter was defined as all billings per patient, per physician, per day. These datasets were linked using unique encoded identifiers and analyzed at ICES.

All children identified by NBS as carriers of the HbS, HbC, HbD, or HbE variants within the timeframe of interest were included in the analyses, and screen negative children were matched at a 5:1 ratio by neighbourhood of residence at birth and by year and month of birth. Because ethnicity is associated with both carrier status and health services use [28, 29], it was an important potentially confounding factor in this study. However, ethnicity is difficult to define [30] and cannot be reliably ascertained using data available at ICES. There is some evidence to suggest that hemoglobinopathy mutations cluster geographically in ethnic communities [31]. Thus, the matching on neighbourhood of residence at birth was designed to partially address confounding by ethnicity. We used an algorithm to individually match children from the screen negative group by forward sortation area (FSA i.e., first three digits of the postal code). In addition, to account for possible confounding related to seasonality or temporal trends in health services use, children from the carrier and screen negative groups were matched by year and month of birth.

Additional potentially confounding variables were adjusted for in our multivariable regression analysis and included: sex, gestational age, and birth weight, all sourced from the CIHI-DAD; and socioeconomic status (by income quintile), based on location of a child’s residence at birth. Specifically, a proxy indicator of socioeconomic status was generated using postal code at the time of a child’s birth linked with Statistics Canada information describing income quintiles by neighbourhood [32]. Children in the lowest two income quintiles were categorized as ‘lower income’ while those in the higher three quintiles were categorized as ‘higher income’.

### Analysis

Descriptive and chi square statistics were generated to describe and compare characteristics between groups. Overall and age-stratified rates of health services use were calculated using person-year denominators to account for different lengths of follow-up. Unadjusted incidence rate ratios (IRRs) with 95% confidence intervals were first generated in a bivariate analysis to compare rates of health services use between children identified as Hb carriers (by carrier type and, separately, by age) and the matched cohort of those who screened negative. To account for potentially confounding factors not addressed through matching, we then compared health services use in children identified as carriers versus matched controls using multivariable regression analysis, stratified by age (< 1 or ≥ 1 year of age). To ensure the analysis was robust in the presence of potential over-dispersion, we used negative binomial regression. The response was the count of health services encounters, the main exposure was carrier status, and an offset term of total person-time at risk was included. Covariates for sex, preterm birth (< or ≥ 37 weeks gestation), low birth weight (< or ≥ 2500 g) and socioeconomic status (lower vs. higher income quintiles) were also included in the model.

### Data sharing

The data that support the findings of this study are housed at ICES and strong restrictions apply to their availability due to privacy regulations.

## Results

### Description of study cohort

**Fig. 1:**
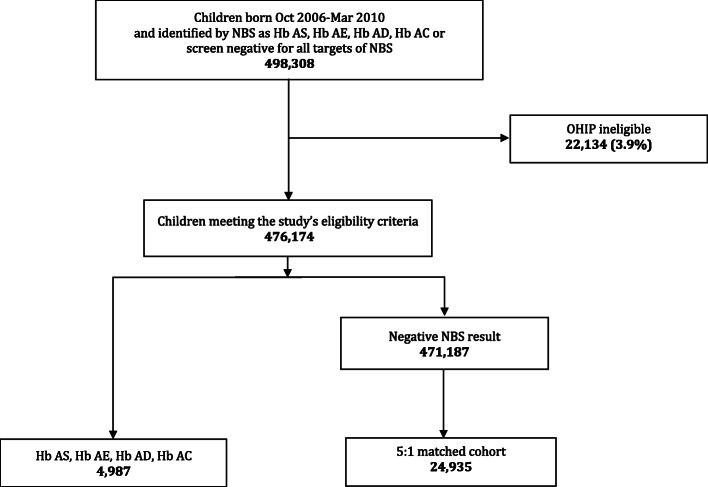
Flow diagram of children who underwent NBS for hemoglobinopathies in Ontario, October, 2006 – March, 2010

Of 476,174 eligible children, 4987 carriers of HbS, HbC, HbD or HbE were identified through NBS and matched to 24,935 study-eligible NBS negative children from the population (Fig. 1). Total follow-up time in the carrier cohort was 18,900 person-years (mean: 3.79 [range: 0.01–5.47]) versus 94,427 person-years (mean: 3.79 [range: 0.002–5.50]) among the control group. There were no statistically significant differences in birth characteristics or socioeconomic status (SES) between hemoglobinopathy carriers and controls (Table 1). Over 99.5% of both cohorts survived to the end of the follow-up period, with no statistically significant differences in mortality.

**Table 1:** Demographic characteristics, Ontario birth cohort Apr 2006- Nov 2012, children with carrier status for HbS, HbC, HbD, or HbE versus children with screen negative newborn screening results

	Carriers *N* = 4987 n (%)	Matched population controls *N* = 24,935 n (%)
Sex^a^		
Male	2472 (50)	12,646 (51)
Female	2515 (50)	12,289 (49)
Birth weight^a^		
< 2500 g	410 (8)	1665 (7)
≥ 2500 g	4549 (92)	23,138 (93)
Gestational age^a^		
< 37 weeks	428 (9)	1920 (8)
≥ 37 weeks	4490 (91)	22,433 (92)
Socioeconomic status^a^		
‘Lower’	2992 (60)	13,679 (55)
‘Higher’	1960 (40)	11,220 (45)
Survival (to end-study)		
Yes	4975 (99.8)	24,867 (99.7)
No	12 (0.2)	68 (0.3)

^a^ Counts for some groups do not add to the total sample size due to missing values

### Health service use outcomes: ED visits

**Fig. 2:**
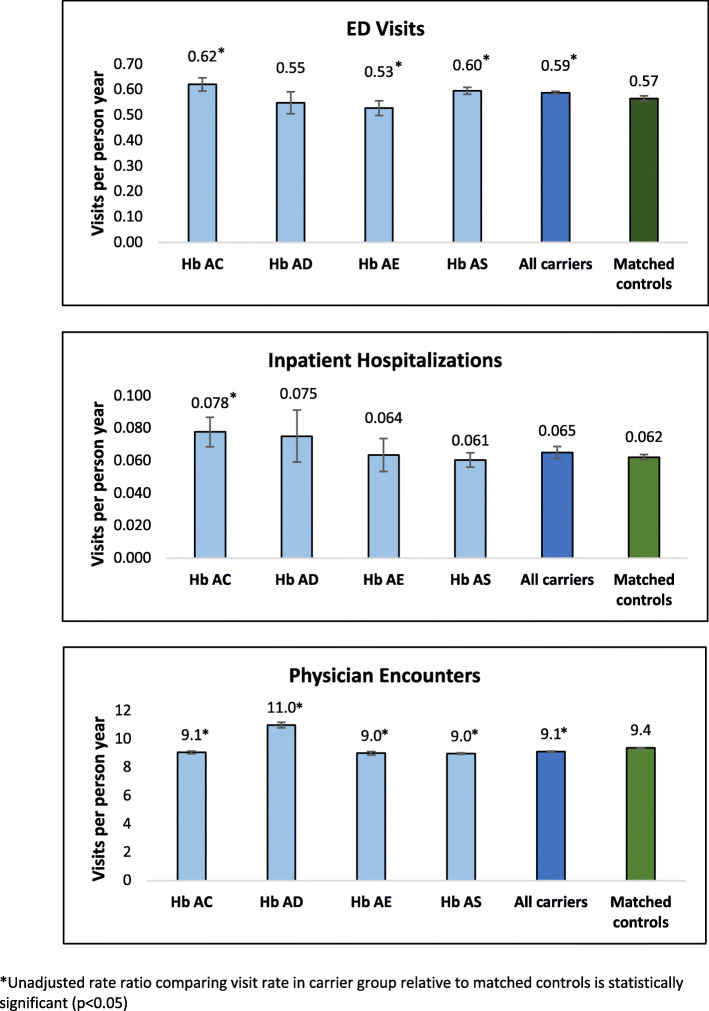
Unadjusted health service utilization rates (average visit rate per child per year) among children identified as Hb carriers through NBS and screen negative matched population controls. Hb carriers presented by Hb variant

There were 11,139 ED visits observed among hemoglobinopathy carriers and 53,386 among screen negative matched controls. The average rate of ED visits per child per year among all hemoglobinopathy carriers was 0.59, ranging from 0.53 (Hb AE) to 0.62 (Hb AC) depending on the specific Hb variant (Fig. 2). The average rate of ED visits per child per year among screen negative matched controls was 0.57. Hb AS carriers showed a small but statistically significant higher rate of ED visits compared to matched controls, as did the Hb AC and “all carriers” groups; the Hb AE carrier group showed a small but statistically significant lower rate of ED visits relative to matched controls. The three most common main diagnoses at the time of the ED visit among both carriers and controls were respiratory infection, fever and ear infection.

**Fig. 3:**
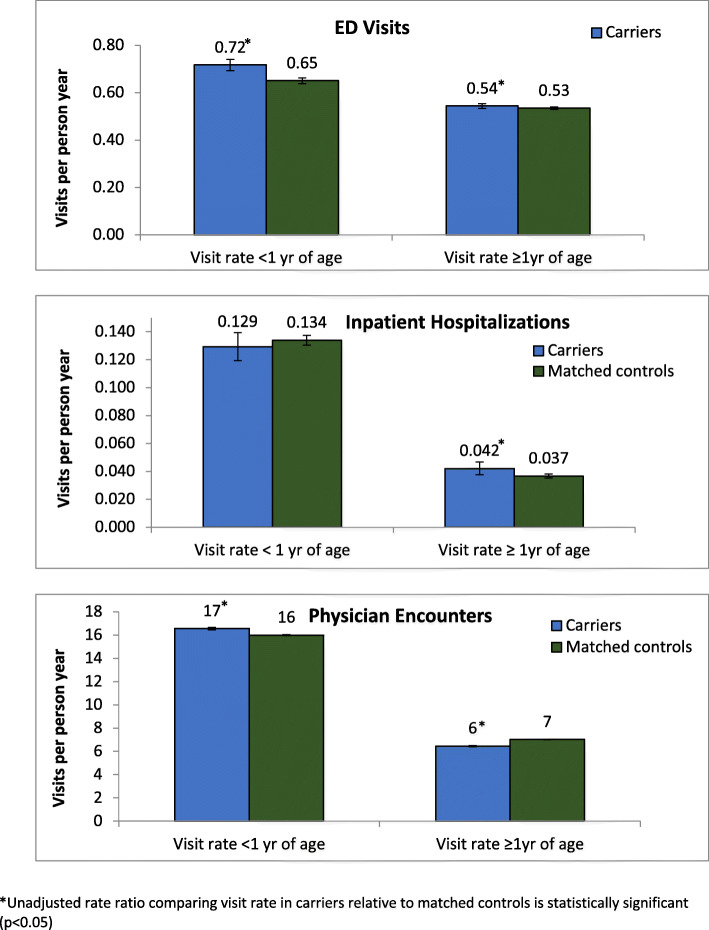
Unadjusted health service utilization rates (average visit rate per child per year) in children identified as Hb carriers through NBS and screen negative matched population controls by age at the time of the visit

By age, for younger children (< 1 year of age), the average rate of ED visits per child per year was 0.72 for carriers and 0.65 for screen-negative controls (Fig. 3), and the unadjusted IRR (95% CI) for ED visits in younger children was 1.10 (1.06–1.14). For older children, ≥1 year of age, the average rate of ED visits per child per year was 0.54 for carriers and 0.53 for controls, with an unadjusted IRR (95% CI) of 1.02 (1.00–1.05). Following adjustment in the regression analysis, a weak, though statistically significant, association was observed between carrier status and increased emergency department visits in infants < 1 year of age: adjusted IRR (aIRR) 1.11 (1.06–1.17). However, this difference was not sustained among older children: aIRR 1.03 (0.98–1.07) (Table 2).

**Table 2:** Adjusted incidence rate ratios, ED visits, inpatient hospitalizations, and physician encounters, for children identified as hemoglobinopathy carriers through NBS and screen negative matched population controls

	ED Visits		Inpatient hospitalizations		Physician encounters	
	< 1 year of age	≥1 year of age	< 1 year of age	≥1 year of age	< 1 year of age	≥1 year of age
**NBS result**						
Carrier	1.11 (1.06–1.17)	1.03 (0.98–1.07)	0.97 (0.89–1.06)	1.14 (1.03–1.25)	1.02 (1.00–1.04)	0.92 (0.90–0.94)
Control	REF	REF	REF	REF	REF	REF
**Sex**						
Male	1.18 (1.14–1.23)	1.20 (1.16–1.23)	1.34 (1.26–1.44)	1.28 (1.19–1.39)	1.09 (1.08–1.11)	1.10 (1.08–1.12)
Female	REF	REF	REF	REF	REF	REF
**Gestational age**						
< 37 weeks	1.33 (1.23–1.45)	1.21 (1.13–1.30)	2.54 (2.27–2.82)	1.36 (1.17–1.57)	1.59 (1.54–1.63)	1.06 (1.02–1.09)
≥ 37 weeks	REF	REF	REF	REF	REF	REF
**Birth weight**						
< 2500 g	1.05 (0.96–1.15)	1.07 (0.99–1.15)	2.03 (1.81–2.28)	1.68 (1.44–1.96)	1.72 (1.67–1.77)	1.15 (1.11–1.20)
≥ 2500 g	REF	REF	REF	REF	REF	REF
**Socioeconomic status**						
‘Lower’	1.17 (1.12–1.21)	1.07 (1.04–1.10)	1.05 (0.99–1.13)	1.05 (0.97–1.14)	1.00 (0.99–1.01)	0.97 (0.96–0.99)
‘Higher’	REF	REF	REF	REF	REF	REF

### Hospital admissions

A total of 7106 hospital admissions were observed across the entire cohort; 1230 occurred among the carrier group, and 5876 among screen negative matched population controls. The average rate of inpatient hospitalizations per child per year among all hemoglobinopathy carriers was 0.065, ranging from 0.061 (Hb AS) to 0.078 (Hb AC) (Fig. 2). The average rate of inpatient hospitalizations per child per year among screen negative controls was 0.062. Only the Hb AC group showed a small but statistically significant difference (higher hospitalization rate) compared to matched controls; the Hb AS group did not. Among carriers, the three most common main diagnoses associated with hospital stays (% visits) were: allergic asthma [6], pneumonia [5], and low birth weight [5]. The most common diagnoses during hospitalization for screen negative controls (% visits) were: neonatal jaundice [6], acute bronchiolitis [5] and pneumonia [5].

For children < 1 year of age, the average rate of hospitalizations per child per year was 0.129 for carriers and 0.134 for controls (Fig. 3), producing an unadjusted IRR (95% CI) of 0.97 (0.89–1.05). In older children ≥1 year of age, the average rate of inpatient hospitalizations per child per year was 0.042 for carriers and 0.037 for screen negatives (Fig. 3), resulting in an unadjusted IRR (95% CI) of 1.15 (1.05–1.26). Following adjustment for potential confounders, infants < 1 year of age who were hemoglobinopathy carriers were generally found to have rates of hospitalization similar to matched population controls with an aIRR (95% CI) of 0.97 (0.89–1.06). We did observe a statistically significant difference among older children following adjustment, whereby children who were carriers had a higher inpatient hospitalization rate compared to those screen negative controls: aIRR (95% CI) 1.14 (1.03–1.25) (Table 2).

### Physician encouters

We observed 172,168 physician encounters among children who were hemoglobinopathy carriers, and 885,674 physician encounters among screen negative matched controls. The average rate of physician encounters per child per year among all hemoglobinopathy carriers was 9.1, ranging from 9.0 (Hb AE, Hb AS) to 11.0 (Hb AD) (Fig. 2). The average rate of physician encounters per child per year among screen negative matched controls was 9.4. Hb S carriers showed a small but statistically significant lower rate of physician encounters compared to matched controls, as did all the other carrier groups with the exception of the Hb AD group, which showed a small but statistically significant higher rate of encounters.

Age-stratified, averaged rates of physician encounters per person-year among children < 1 year were 17 for carriers and 16 for controls (Fig. 3). This resulted in an unadjusted IRR (95% CI) of 1.03 (1.03–1.04) among younger children. In the older group, the average rate of physician encounters per child per year was 6 for carriers and 7 for controls, with an unadjusted IRR (95% CI) in children ≥1 year of age of 0.92 (0.91–0.94). Following adjustment, rates of physician encounters in younger children who were carriers were similar to controls with an aIRR (95% CI) of 1.02 (1.00–1.04) (Table 2). In children ≥1 year of age a statistically significantly lower rate of physician encounters was observed in carriers as compared to matched controls with an aIRR (95% CI) of 0.92 (0.90–0.94).

## Discussion

Understanding the implications of hemoglobinopathy carrier status in newborns and young children is important for informing the development and implementation of NBS policy governing the disclosure of carrier status results to families when they are identified incidentally through screening for sickle cell disease. In this longitudinal study of children in Ontario, where NBS carrier results for hemoglobinopathy mutations (i.e., Hb AS, Hb AC, Hb AD, Hb AE) are not disclosed to families by default, children who were carriers of such mutations did not consistently use health services at different rates than matched population controls who screened negative. We did observe some statistically significant differences in the two groups. Although some Hb carrier types had health services use rates that were statistically significantly different relative to matched controls in the bivariate analysis (Fig. 2), the magnitude of these differences were small and there were no clear patterns pointing to clinically important differences in health service use. Specifically, HbAS children had statistically significant but only slightly higher rates of ED visits and lower rates of physician visits, as well as a lower rate of hospitalization that was not statistically different. Further, in the multivariable analysis hemoglobinopathy mutation carriers experienced a 1.11 times higher frequency of ED visits compared to the matched control group at less than 1 year of age, but there was no statistically significant difference in the groups in ED visits among older children. Conversely, carriers who were 1 year of age or older experienced a higher (1.14 times the frequency) rate of inpatient hospitalizations and a lower (0.92 times the frequency) rate of physician encounters compared to controls, but neither association was observed among infants under 1 year of age.

Thus, the statistically significant associations in our study were all relatively weak, were not consistent across health service types or age groups, and the direction of associations was not consistent. For example, some observed effects in the multivariable models suggested higher utilization by carriers (higher hospitalization > = 1 year of age and higher ED use at < 1 year of age), while other suggested lower utilization (lower physician contacts > = 1 year of age). The higher hospitalization rate we observed among carriers over 1 year of age is a potentially concerning finding, since hospitalization is a relatively robust outcome as an indicator of poor health. However, while significant concern and debate have been raised concerning sickle cell carrier status and a risk of acutely adverse health outcomes, most often in high-performing athletes or other strenuous circumstances [33, 34], these concerns appear to be limited to adults [10, 35], and the nature and extent of the risk is uncertain [13–16]. The only comparable population-based data in children are from Reeves and colleagues, who found no evidence of elevated ED visit rates nor hospitalizations among children with sickle cell trait identified by newborn screening in Michigan, relative to children with normal hemoglobin newborn screening results [17]. Therefore, we consider that the most likely explanations for the higher hospitalization rate we observed among carriers over 1 year of age in our study are chance (given the small effect size) or residual confounding. With respect to the latter, although we matched on neighbourhood, birth year, and birth month and adjusted for SES and birth characteristics, we were unable to directly adjust for ethnicity, which is strongly associated with the presence of hemoglobinopathy mutations and has also been demonstrated to be associated with both health outcomes and access to health services. For example, Quan and colleagues describe a population-based study using data from the Canadian Community Health Survey and report disparities in health services use by ethnicity [28]. Likewise, Wu and colleagues report higher unmet health care needs and different patterns of use among more-recent immigrants to Canada [29].

The higher rate of ED visits among carriers under the age of one year relative to controls may, similarly, be due to chance or residual confounding. While parents who know that their child is a carrier may visit the ED more frequently as a result of concern about the carrier result, Ontario’s NBS disclosure policy and the small number of families who have proactively sought carrier status information suggest that this is not a plausible explanation for the observed increase in our study. It is unclear why children who were carriers over the age of one year experienced a lower rate of physician encounters relative to population controls. This finding in particular points to the possible role of chance.

While our results do not directly inform policy considerations for disclosure of sickle cell carrier results ascertained by newborn screening, they are relevant. Previous work has suggested that parents would prefer to receive carrier results (e.g. 20 and 24) and health care providers generally feel a duty to disclose such results [36]. If our study had detected important differences in health care utilization between carriers and non-carriers, one could expect that the possibility of morbidity associated with carrier status might have led to increased parental interest in receiving result. Further, it would have been important to revisit the current Ontario carrier disclosure policy in that context. However, as no differences in health care utilization were detected in our study, it is unlikely that our findings will impact parental interest in receiving carrier results.

### Strengths and limitations

Routinely-collected health care administrative datasets in Ontario allow for population-based research with near complete population coverage of a population in excess of 14 million people, and over 140,000 live births per year. Given this powerful feature, these studies are particularly useful in the context of research into rare diseases, conditions and exposures where sufficient sample sizes are challenging to achieve. Our study was therefore able to describe longitudinal health services use among children with hemoglobinopathy carrier status as compared to a matched population control group.

Nonetheless, the breadth of investigation that routinely-collected health care administrative datasets allow for is offset by several limitations. In general, because these large datasets were not designed for research purposes, they contain artefacts and features that can make interpretation challenging. For example, physician billings described in Ontario’s OHIP data do not exclude services rendered in the ED and in hospital. Thus, some physician encounters described in our study overlap with some ED visits and inpatient hospital admissions. Nonetheless, there is no reason to believe that this would have introduced a differential bias, and it would thus be unlikely to have a meaningful impact on the comparative analysis of health services use. Perhaps the most important limitation for our study was the lack of detailed clinical information and details regarding personal characteristics in the administrative datasets. Notably, ethnicity was an important potential confounding factor and this was not available in the health administrative data. While our efforts to match by neighbourhood of residence may have accounted for this in part, we were not able to completely address confounding related to ethnicity in this study.

Further, since follow up in this study was restricted to the first few years of life, our study does not contribute evidence toward the question of the impact of hemoglobinopathy mutation carrier status on health services use among older children and adults, which would be an important follow up as many clinical outcomes associated with HbS carrier status have been noted in adulthood [10]. The assembly of the current cohort will provide an ongoing opportunity for expansion and longer-term follow up toward an improved understanding of any effects of carrier status on health services use in adulthood.

## Conclusion

Our study did not identify a clear, clinically relevant, association between hemoglobinopathy carrier status identified by NBS and health services utilization. The weakness of the observed associations and their inconsistent direction, combined with the inconsistencies across age groups and types of health services, challenge the importance of the specific statistically significant findings. This lack of a clear association with health services use suggests that the health care needs of children who are carriers of hemoglobinopathy mutations are likely similar to those of the population of children who are not carriers in the first few years of life. Nonetheless, ongoing research is needed to ensure that hemoglobinopathy carrier status does not affect health services use and needs later in life.

## Data Availability

The dataset from this study is held securely in coded form at ICES. While legal data sharing agreements between ICES and data providers (e.g., healthcare organizations and government) prohibit ICES from making the dataset publicly available, access may be granted to those who meet pre-specified criteria for confidential access, available at www.ices.on.ca/DAS (email: das@ices.on.ca). The full dataset creation plan and underlying analytic code are available from the authors upon request, understanding that the computer programs may rely upon coding templates or macros that are unique to ICES and are therefore either inaccessible or may require modification.
